# Correlation between gabapentin and depression: A study from the NHANES and FAERS databases

**DOI:** 10.1097/MD.0000000000044010

**Published:** 2025-08-22

**Authors:** Hao Zhang, Hua Huang, Hongqi Ou, Xi Luo, Ping Zhang, Panli Zhao

**Affiliations:** a Department of Pharmacy, Chengdu Seventh People’s Hospital (Affiliated Cancer Hospital of Chengdu Medical College), Shuangliu District, Chengdu, Sichuan Province, China.

**Keywords:** depression, FAERS, gabapentin, NHANES

## Abstract

Post-marketing surveillance has indicated an association between gabapentin use and an increased risk of depression. However, observational findings on this relationship have been inconsistent. This study aims to investigate the correlation between gabapentin exposure and depression. We analyzed data from the National Health and Nutrition Examination Survey and the Food and Drug Administration Adverse Event Reporting System in the United States from 2011 to 2018. Descriptive statistical analysis, multivariate logistic regression, and linear regression were employed to explore the association between gabapentin use and depression. Our analysis revealed that gabapentin users had a higher risk of depression. In a multivariate logistic regression model, the odds ratio was 1.8 (95% confidence interval: 1.3–2.4; *P* < .001), indicating a significant association when accounting for demographics and lifestyle factors. Similarly, in a linear regression model, the depression score was significantly higher (β = 4.0; 95% confidence interval: 3.0–5.0; *P* < .001) among gabapentin users. This risk was notably greater in women and individuals who slept <7 hours. The Food and Drug Administration Adverse Event Reporting System database included 9951 adverse reactions, with 1165 reports of psychiatric-related adverse events, including depression, constituting 11.71% of the total reports. Gabapentin use is associated with an increased risk of depression. It is crucial for clinicians to monitor patients’ mental health closely when prescribing gabapentin and to provide timely intervention if needed.

## 1. Introduction

Depression is a significant mental health disorder, marked by persistent low mood, loss of interest, low energy, and diminished self-esteem.^[1]^ This disorder not only severely impacts daily functioning but also poses risks of social impairment and suicide.^[2]^ The pathogenesis of depression is multifactorial, involving genetic, environmental, and biological interactions.^[3]^

Gabapentin, a GABA analog approved in 1993, is primarily used to treat partial epilepsy, neuropathic pain, and restless legs syndrome.^[4,5]^ Its mechanism involves modulating calcium (Ca2+) channels by binding to the α2δ subunit in the central nervous system, thereby influencing excitatory neurotransmitter release.^[6,7]^ Beyond its original indications, gabapentin has gained attention post-marketing for its ability to reduce postoperative pain and opioid dependence in perioperative care.^[8]^ Additionally, it has been suggested to aid in reducing alcohol withdrawal symptoms and relapse risk,^[9]^ alleviate chronic pruritus, especially that associated with chronic kidney disease or cholestasis,^[10]^ and relieve menopausal hot flashes in women who cannot use estrogen replacement therapy.^[11]^ Neurologically, gabapentin is used to treat generalized anxiety disorder, social anxiety disorder, and other anxiety-related conditions.^[12]^ It has also shown efficacy in improving sleep quality in patients with insomnia related to anxiety or neuralgia^[13]^ and as an adjunct in the management of bipolar disorder and other mood disorders.^[14]^ Furthermore, gabapentin may be used as an adjunctive therapy in refractory depression to enhance the effects of traditional antidepressants or mitigate their side effects, particularly in patients with significant anxiety symptoms or chronic neuropathic pain, thereby indirectly improving depressive symptoms.^[15–17]^ Despite its tolerability and safety profile, gabapentin has been associated with a growing number of side effects, including common issues such as dry mouth, constipation, diarrhea, dizziness, somnolence, and ataxia.^[18]^ More severe adverse effects include respiratory depression,^[19]^ sexual dysfunction,^[20]^ rhabdomyolysis,^[21]^ worsening of anxiety and depression, hallucinations, psychosis,^[22]^ and suicidal behavior.^[23]^

The National Health and Nutrition Examination Survey (NHANES) is a key health and nutrition assessment program in the United States, providing a comprehensive view of the nation’s health through questionnaires and physiological measurements in representative samples. It offers valuable insights into population health, nutritional intake, and chronic disease prevalence.^[24]^ In contrast, the U.S. Food and Drug Administration’s Adverse Event Reporting System (FAERS) is a critical drug safety monitoring database that records adverse events reported by healthcare professionals and patients worldwide, reflecting real-world drug safety and risks.

This study aims to systematically evaluate the relationship between gabapentin use and depression by analyzing data from NHANES and FAERS. Our goal is to deepen the understanding of gabapentin’s potential effects on depressive symptoms across different populations and provide empirical support for clinical decision-making and medication management.

## 2. Materials and methods

### 2.1. Data sources

Data were derived from the NHANES, a nationally representative survey conducted by the National Center for Health Statistics. Participants were selected using a multi-stage, stratified probability sampling strategy. Data collection involved comprehensive household interviews, physical examinations, and blood sample analysis conducted at Mobile Examination Centers. The study utilized NHANES data from 2011 to 2018 to maintain consistency in the definition of covariates, and all data were obtained from the official NHANES website.^[25]^ Adverse event data were extracted from OpenVigil 2.1, an online pharmacovigilance data mining tool widely used in safety studies.^[26]^ We queried the tool for adverse drug events (ADEs) associated with gabapentin from January 1, 2011 to December 31, 2018.

### 2.2. Exposure assessment

Gabapentin exposure was assessed based on participants’ self-reported prescription drug use, with the question: “Have you taken or used any prescription drugs in the past month?” Respondents who answered affirmatively were classified as the exposed group, while those who did not use gabapentin were classified as the control group. The Prescription Drug Questionnaire also captured the duration of gabapentin use with the question: “How long have you been using or taking (product name)?” To align with the depression assessment window of the Patient Health Questionnaire (PHQ-9), participants who had used gabapentin for <14 days were excluded.

### 2.3. Assessment of depressive symptoms

Depressive symptoms were evaluated using the PHQ-9 scale as part of the Mobile Examination Center assessment. The PHQ-9 consists of 9 items, each scored from 0 (not at all) to 3 (nearly every day), resulting in a total score range of 0 to 27. Higher scores indicate more severe depressive symptoms,^[27]^ with scores of 10 or greater suggesting the presence of major depression.^[28]^

### 2.4. Covariates

We assessed a variety of potential covariates, including age, gender, race/ethnicity, education, poverty-to-income ratio, body mass index, smoking status, energy intake, caffeine intake, sugar intake, alcohol intake, diabetes mellitus, hypertension, physical activity, and sleep duration. Age was treated as a continuous variable. Race/ethnicity was self-reported and categorized as Mexican American, other Hispanic, non-Hispanic White, non-Hispanic Black, or other. Educational attainment was classified as less than high school, high school graduate or equivalent, some college, and college graduate or higher. Poverty-to-income ratio was calculated as the ratio of household income to the poverty threshold, ranging from 0 to 5. Smoking status was categorized as never, current, or former smokers. Physical activity was assessed based on responses to whether participants engaged in moderate-intensity activities. Body mass index was calculated by dividing weight (kg) by height (m²). Sleep duration was derived from self-reported hours of sleep on weekdays. Diabetes and hypertension were determined based on self-reports of physician diagnoses.^[28–30]^

### 2.5. Statistical analysis

Given the complex survey design of NHANES, statistical analyses incorporated sample weights, clustering, and stratification. Continuous variables are presented as means and categorical variables as frequencies (%). Logistic and linear regression models were used to estimate odds ratios (OR) or beta coefficients (β) with 95% confidence intervals (CIs) for the association between gabapentin use and depression. Model 1 was unadjusted, Model 2 adjusted for age, sex, race, education, and income, and Model 3 was fully adjusted, additionally accounting for physical activity, sleep duration, diabetes, hypertension, energy intake, caffeine intake, alcohol intake, and sugar intake. Interaction and subgroup analyses were conducted across various demographic and clinical factors. Sensitivity analyses included redefining depression using a PHQ-9 score ≥ 10, treating PHQ-9 scores as a continuous variable, and evaluating predictive power using receiver operating characteristic (ROC) curves.^[31]^ Additionally, we explored real-world ADE data from FAERS using reporting odds ratios (RORs) and Bayesian Confidence Propagation Neural Networks.^[32]^ Signal detection thresholds for ADEs were defined based on both algorithms, with a signal indicating a potential drug event association shown in Tables 1 and 2. Statistical analyses were performed using R version 4.4.0 (Posit, Boston) and Microsoft Excel (Redmond), with significance set at *P* < .05.

**Table 1:** Four-fold table for calculation.

	Topotecan	Non-Topotecan
Target AEs	a	c
Non-target AEs	b	d
N = a + b + c + d		

AEs = adverse events.

**Table 2:** Formulas and thresholds of ROR and BCPNN.

Method	Formula	Threshold
ROR	ROR = $\frac{a/c}{b/d}$ 95% CI = e^($\ln ROR\pm 1.96\sqrt{1/a+1/b+1/c+1/d}$)	a ≥ 3 and 95 % CI (lower limit) > 1
BCPNN	IC = $\log_{2}\frac{a\left(a+b+c+d\right)}{\left(a+b\right)\left(a+c\right)}$ $\gamma =\gamma_{ij}\frac{\left(N+\alpha \right)\left(N+\beta \right)}{\left(a+b+\alpha_{i}\right)\left(a+c+\beta_{j}\right)}$ $E\left(IC\right)=\log_{2}\frac{\left(a+\gamma_{ij}\right)\left(N+\alpha \right)\left(N+\beta \right)}{\left(N+\gamma \right)\left(a+b+\alpha_{i}\right)\left(a+c+\beta_{j}\right)}$ $V\left(IC\right)={\left(\frac{1}{\ln2}\right)}^{2}\left(\frac{N-\alpha +\gamma -\gamma_{ij}}{\left(\alpha +\gamma_{ij}\right)\left(1+N+\gamma \right)}+\frac{N-a-b+\alpha -\alpha_{i}}{\left(a+b+\alpha_{i}\right)\left(1+N+\alpha \right)}+\frac{N-a-c+\beta -\beta_{j}}{\left(a+c+\beta_{j}\right)\left(1+N+\beta \right)}\right)$ $SD=\sqrt{V\left(IC\right)}$ IC025 = E(IC)−2SD	IC025 > 0

BCPNN = Bayesian Confidence Propagation Neural Networks, CI = confidence interval, ROR = reporting odds ratio.

## 3. Results

### 3.1. Participant characteristics

**Figure 1.**
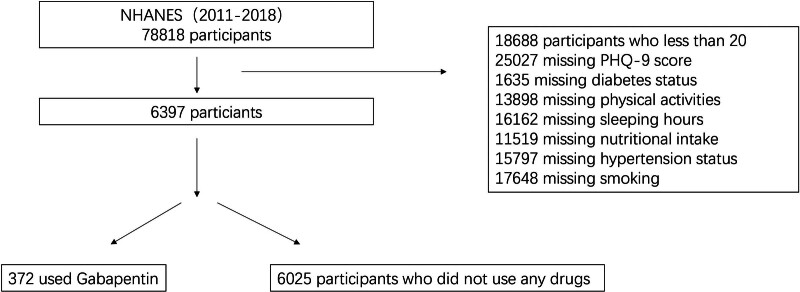
Flowchart of the sample selection from NHANES. NHANES = National Health and Nutrition Examination Survey.

A total of 78,818 participants were enrolled in NHANES from 2011 to 2018. After applying exclusion criteria: including age under 20 years, pregnancy, missing PHQ-9 data, lack of medication use, incomplete nutrient intake data, missing information on hypertension, diabetes mellitus, smoking status, and physical activity: a final cohort of 6397 participants was included in this analysis. Of these, 372 individuals reported gabapentin use, while 6025 did not use any medication. Among the study population, 419 participants were identified as having depression, and 5978 were classified as non-depressed. Key demographic and lifestyle characteristics revealed that women had a higher prevalence of depression compared to men. Furthermore, individuals who did not engage in regular physical activity exhibited a 67% higher risk of developing depression. Additionally, those with depression reported higher rates of smoking and caffeine intake. Detailed demographic and lifestyle information is presented in Figure 1 and Table 3.

**Table 3:** Baseline characteristics of the study participants (n = 6397).

Characteristic	N*	Overall, N = 70,738,008†	No depression, N = 66,601,031†	Depression, N = 4,136,977†	*P*-value‡
Age	6397	40 ± (14)	40 ± (14)	43 ± (16)	.034
Gender	6397				<.001
Male		3630 (57%)	3454 (59%)	176 (39%)	
Female		2767 (43%)	2524 (41%)	243 (61%)	
Race	6397				.014
Mexican American		1070 (12%)	1017 (12%)	53 (7.8%)	
Other Hispanic		699 (7.8%)	633 (7.6%)	66 (12%)	
Non-Hispanic White		2084 (59%)	1926 (59%)	158 (57%)	
Non-Hispanic Black		1372 (12%)	1278 (11%)	94 (14%)	
Other race		1172 (9.8%)	1124 (9.8%)	48 (9.7%)	
Education level	6397				<.001
High school graduate or below		2794 (38%)	2557 (37%)	237 (52%)	
Some college		1988 (32%)	1857 (32%)	131 (33%)	
College graduate or above		1615 (30%)	1564 (31%)	51 (15%)	
Income	6397	2.82 ± (1.65)	2.87 ± (1.65)	1.94 ± (1.52)	<.001
BMI	6397	28.0 ± (4.7)	27.9 ± (4.7)	29.5 ± (5.2)	<.001
Energy	6397	2299 ± (1047)	2308 ± (1041)	2156 ± (1143)	.004
Sugar	6397	116 ± (80)	115 ± (79)	130 ± (90)	.032
Caffeine	6397	164 ± (212)	161 ± (204)	212 ± (314)	.2
Alcohol	6397	14 ± (35)	14 ± (34)	12 ± (41)	<.001
Sleep hours	6397	7.21 ± (1.37)	7.23 ± (1.34)	6.97 ± (1.80)	.008
Smoking	6397				<.001
Yes		2552 (41%)	2320 (40%)	232 (56%)	
No		3845 (59%)	3658 (60%)	187 (44%)	
Hypertension	6397				<.001
Yes		919 (13%)	775 (12%)	144 (33%)	
No		5478 (87%)	5203 (88%)	275 (67%)	
Moderate activities	6397				<.001
Yes		2793 (47%)	2661 (48%)	132 (33%)	
No		3604 (53%)	3317 (52%)	287 (67%)	
Diabetes	6397				<.001
Yes		264 (2.9%)	213 (2.4%)	51 (11%)	
No		6133 (97%)	5765 (98%)	368 (89%)	
PHQ-9 score	6397	3 ± (4)	2 ± (2)	14 ± (4)	<.001
Gabapentin	6397				<.001
No		6025 (95%)	5721 (96%)	304 (74%)	
Yes		372 (5.2%)	257 (3.9%)	115 (6%)	

BMI = body mass index, CI = confidence interval, PHQ-9 = Patient Health Questionnaire.

* N not missing (unweighted).

† Mean ± (SD); n (unweighted) (%).

‡ Wilcoxon rank-sum test for complex survey samples; chi-squared test with Rao & Scott second-order correction.

### 3.2. Logistic regression results

We evaluated the association between gabapentin use and depression through 3 logistic regression models. Model 1, which was unadjusted for any covariates, indicated that gabapentin users had a 120% increased odds of experiencing depression compared to non-users, with an OR of 2.2 (95% CI: 1.8–2.6; *P* < .001). In Model 2, which adjusted for demographic variables including age, gender, race/ethnicity, education level, and income, the OR decreased to 1.8 (95% CI: 1.3–2.4; *P* < .001). Model 3, which further adjusted for all covariates from Model 2, maintained a significant association between gabapentin use and depression. The detailed results are summarized in Table 4.

**Table 4:** Association between taking gabapentin and depression.

(OR (95% CI) *P*-value)	Model 1	Model 2	Model 3
Gabapentin			
0	Ref	Ref	Ref
1	2.2 (1.8, 2.6) < .001	2.2 (1.7, 2.7) < .001	1.8 (1.3, 2.4) < .001

CI = confidence interval.

### 3.3. Interaction and subgroup analyses

**Figure 2.**
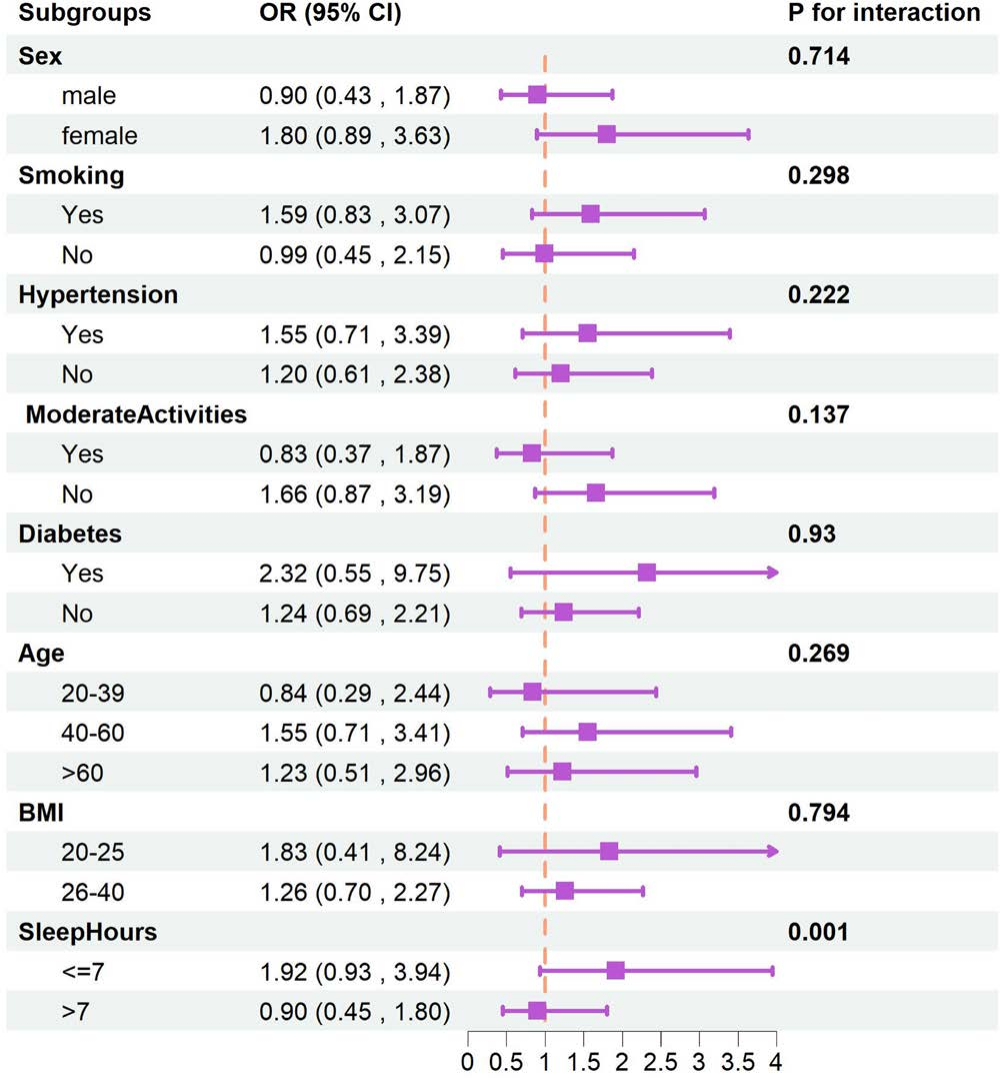
The subgroup analysis.

We conducted interaction and subgroup analyses to explore how various covariates might influence the association between gabapentin use and depression. The analyses were stratified by gender, age, body mass index, smoking status, hypertension, physical activity, diabetes mellitus, and sleep duration. Among these factors, only sleep duration showed a statistically significant interaction with gabapentin use, with a *P*-value < .05. No other covariates demonstrated a significant interaction effect, as indicated by *P*-values > .05. These findings are detailed in Figure 2.

### 3.4. Sensitivity analysis

To assess the robustness of our findings, we conducted several sensitivity analyses. First, we performed population-specific logistic regression analyses with gabapentin use as the exposure and depression as the outcome, stratified by gender and sleep duration. The models were adjusted for the same covariates as the original analysis. The results indicated that, after full adjustment, the OR for males was 1.6 (95% CI: 0.85–2.4; *P* < .001), and for females, it was 2.0 (95% CI: 1.2–2.7; *P* < .001). Additionally, individuals with <7 hours of sleep per night had a higher risk of depression, as detailed in Table 5. Second, we conducted multivariate linear regression analyses with the PHQ-9 score as a continuous outcome, also examining effects stratified by gender and sleep duration. Gabapentin exposure showed a significant correlation with the severity of depressive symptoms. Specifically, the association was more pronounced in women, with a β of 4.6 (95% CI: 3.2–6.0; *P* < .001), and in individuals with <7 hours of sleep, with a β of 4.6 (95% CI: 3.4–5.8; *P* < .001). These results align with the logistic regression findings and are further detailed in Table 6.

**Table 5:** Association between taking gabapentin and depression in gender and sleep hours status.

(OR (95% CI) *P*-value)	Model 1	Model 2	Model 3
Male			
0	Ref	Ref	Ref
1	1.9 (1.3, 2.4) < .001	1.8 (1.1, 2.5) < .001	1.6 (0.85, 2.4) < .001
Female			
0	Ref	Ref	Ref
1	2.2 (1.7, 2.7) < .001	2.4 (1.8, 3.1) < .001	2.0 (1.2, 2.7) < .001
Sleep hours ≤ 7			
0	Ref	Ref	Ref
1	2.5 (2.0, 2.9) < .001	2.3 (1.7, 2.9) < .001	2.0 (1.3, 2.7) < .001
Sleep hours>7			
0	Ref	Ref	Ref
1	1.8 (1.3, 2.3) < .001	2.2 (1.5, 2.8) < .001	1.7 (1.1, 2.4) < .001

CI = confidence interval.

**Table 6:** Association between taking gabapentin and PHQ-9 score.

(β (95% CI) *P*-value)	Model 1	Model 2	Model 3
Gabapentin			
0	Ref	Ref	Ref
1	4.6 (3.7, 5.5) < .001	4.6 (3.7, 5.6) < .001	4.0 (3.0, 5.0) < .001
Male			
0	Ref	Ref	Ref
1	3.4 (2.6, 4.2) < .001	3.5 (2.6, 4.3) < .001	3.1 (2.2, 4.0) < .001
Female			
0	Ref	Ref	Ref
1	5.3 (4.0, 6.6) < .001	5.5 (4.2, 6.8) < .001	4.6 (3.2, 6.0) < .001
Sleep hours ≤ 7			
0	Ref	Ref	Ref
1	5.5 (4.5, 6.6) < .001	5.3 (4.2, 6.5) < .001	4.6 (3.4, 5.8) < .001
Sleep hours>7			
0	Ref	Ref	Ref
1	3.6 (2.4, 4.9) < .001	4.0 (2.8, 5.2) < .001	3.5 (2.4, 4.7) < .001

CI = confidence interval, PHQ-9 = Patient Health Questionnaire.

### 3.5. ROC analysis

**Figure 3.**
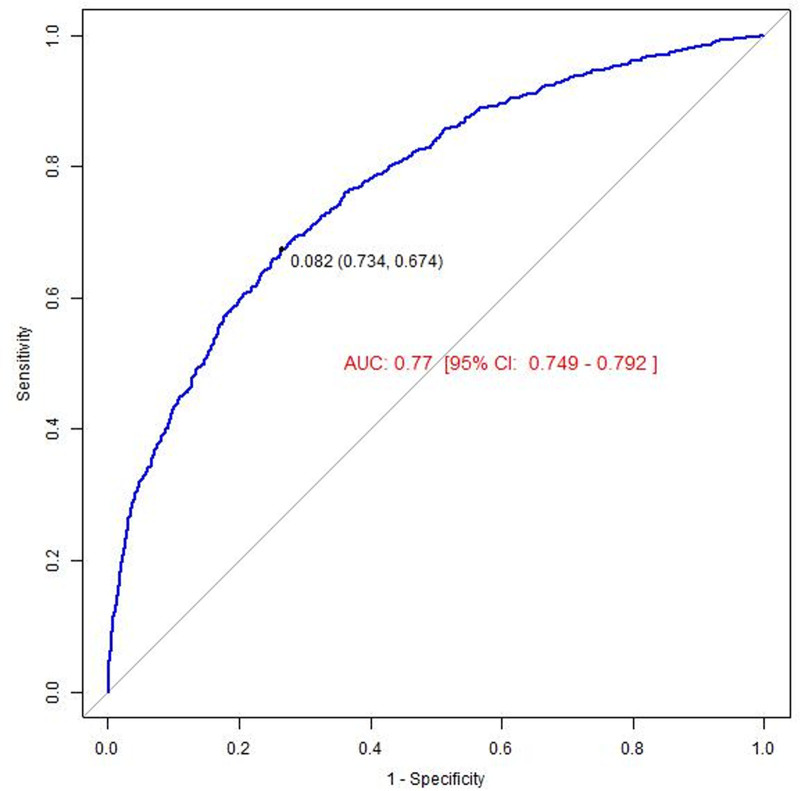
The result of receiver operator curve (ROC).

Following adjustment for all covariates, the area under the ROC for predicting the risk of depression associated with gabapentin exposure was 0.780 (95% CI: 0.756–0.804). This area under the ROC value indicates a reliable predictive performance for the outcome of interest. Detailed results are presented in Figure 3.

### 3.6. FAERS database results

**Figure 4.**
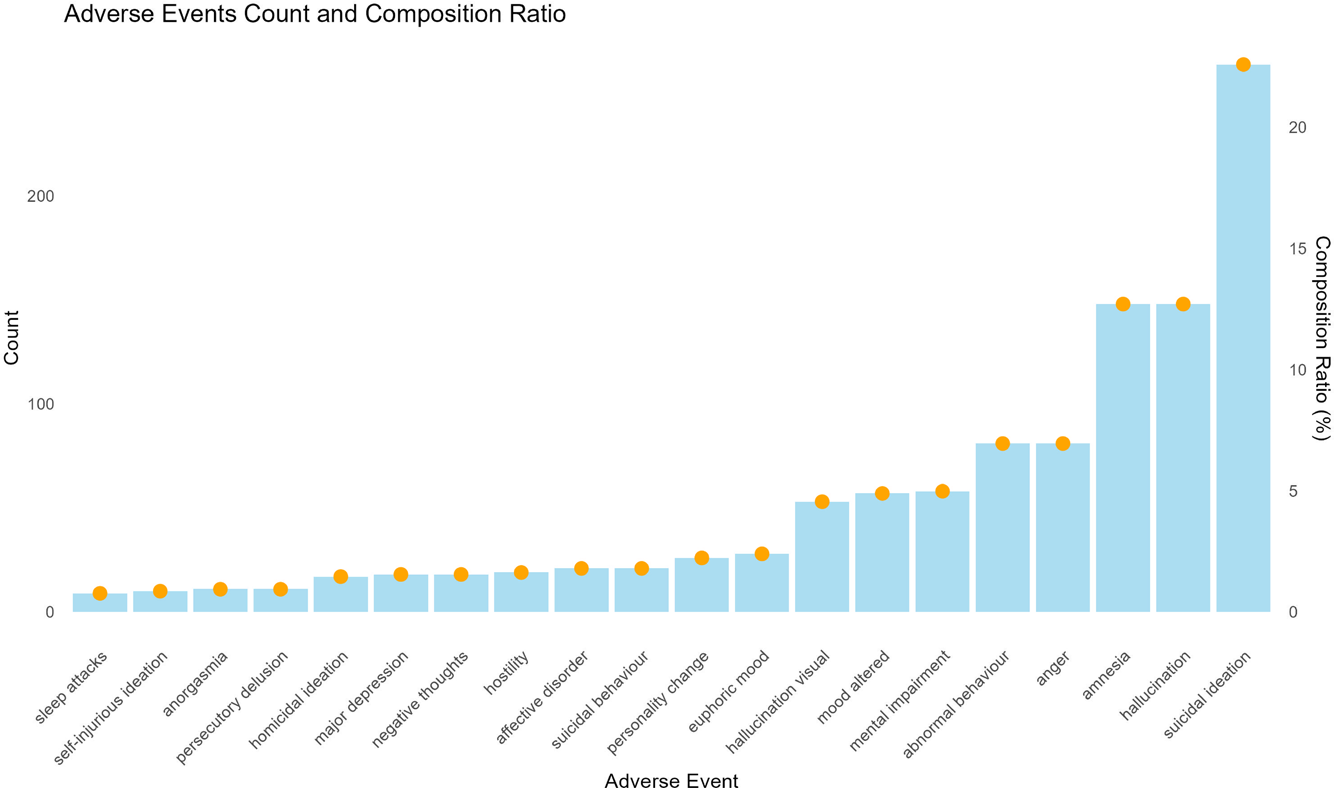
Adverse events count and composition ratio.

In the U.S. FAERS, a total of 1165 reports were identified where gabapentin was the primary suspected drug associated with depression-related adverse events. Among these, suicidal ideation was the most frequently reported, with 263 cases (22.58%). The distribution of the top 20 preferred terms by frequency is illustrated in Figure 4. Additionally, the ROR and 95% CI for these events are summarized in Table 7. The highest ROR was observed for insomnia, with a value of 32.03 (95% CI, 15.72–65.31). Suicidal ideation also showed a significant ROR of 4.12 (95% CI, 3.64–4.66). Both findings were statistically significant.

**Table 7:** Top 20 preferred terms of signal intensity.

Adverse event	ROR	95% CI high	95% CI low
Suicidal ideation	4.12	4.66	3.64
Hallucination	3.88	4.57	3.29
Amnesia	3.27	3.85	2.77
Anger	3.35	4.17	2.69
Abnormal behavior	3.32	4.14	2.66
Mental impairment	3.30	4.28	2.54
Mood altered	3.29	4.27	2.53
Hallucination visual	4.63	6.09	3.53
Euphoric mood	3.95	5.74	2.71
Personality change	4.07	6.01	2.76
Affective disorder	4.22	6.51	2.74
Suicidal behavior	5.60	8.65	3.63
Hostility	7.37	11.66	4.65
Negative thoughts	7.56	12.13	4.71
Major depression	3.59	5.73	2.25
Homicidal ideation	4.82	7.81	2.98
Anorgasmia	4.46	8.13	2.45
Persecutory delusion	5.39	9.82	2.95
Self-injurious ideation	3.25	6.09	1.74
Sleep attacks	32.03	65.31	15.72

CI = confidence interval, ROR = reporting odds ratio.

## 4. Discussion

The association between gabapentin use and an increased risk of depression has been a subject of ongoing debate. This study provides a comprehensive analysis of data from the NHANES database (2011–2018) to evaluate the relationship between gabapentin exposure and depression. Our findings indicate that individuals using gabapentin are significantly more likely to develop depression compared to those not taking any medication. Notably, in the interaction and subgroup analyses, only the interaction with sleep duration had a statistically significant *P*-value (<.05), suggesting a strong correlation. Further analysis revealed that women and those with less than 7 hours of sleep per night are at a higher risk of depression. These findings were consistent with those obtained using a linear regression model with PHQ-9 scores as the outcome variable. The study’s predictive model was further validated through ROC curve analysis, demonstrating good reliability. Additionally, real-world evidence from the FAERS database revealed that approximately 10% of reported adverse reactions related to gabapentin involve depression, further supporting the observed association. This relationship persisted even after adjusting for potential confounders, including demographic factors, dietary habits, smoking status, alcohol consumption, the presence of hypertension, diabetes, and physical activity levels.

Gabapentin, a widely prescribed^[33]^ medication with complex mechanisms of action within the nervous system, has attracted considerable interest regarding its potential role in treating depression. Some studies suggest that gabapentin may exert antidepressant effects by inhibiting the release of excitatory neurotransmitters (e.g., glutamate) through its binding to α2δ subunits in the nervous system. This mechanism may alleviate depressive symptoms and promote neural plasticity, which could help repair neurofunctional abnormalities associated with depression.^[34–37]^ However, conflicting evidence suggests that gabapentin is ineffective as a monotherapy for depression^[38]^ and may even exacerbate mood disturbances,^[39]^ particularly with prolonged or high-dose use. These adverse effects might be linked to gabapentin’s inhibitory actions on excitatory neurotransmitters, especially in cases where neuroadaptive processes are not fully understood. Moreover, observational studies have raised concerns about an increased risk of suicidal behavior associated with gabapentin use.^[40]^ While the underlying mechanisms remain unclear, these findings underscore the importance of closely monitoring patients’ psychological well-being during gabapentin treatment, particularly at treatment initiation and during dose adjustments.^[41–43]^ Given the variability in individual responses to gabapentin, clinical decisions should be tailored to the patient’s overall health and specific needs, particularly in those with a history of depression or mood disorders.

## 5. Limitations

This study has several limitations. First, the NHANES database is based on a cross-sectional design, which relies heavily on self-reported data and is observational by nature. Additionally, the exclusion of participants due to missing covariates may have introduced bias. The FAERS database, being a spontaneous reporting system, is prone to issues such as underreporting, misreporting, and missing information, and it aggregates data from diverse sources, including pharmaceutical companies, patients, and healthcare providers, which can introduce reporting bias.^[44]^ Although we employed both the ROR method and the Bayesian Confidence Propagation Neural Networks method to enhance the screening threshold for ADE signals, the possibility of false-positive results cannot be entirely ruled out. Consequently, this study demonstrates an association rather than causality. Furthermore, depression is a multifactorial condition influenced by numerous variables, necessitating further prospective research to corroborate our findings. Due to the lack of detailed medication records in both databases, this study was unable to conduct an in-depth analysis of the gradient relationship between gabapentin dose/treatment duration and depression risk.

## 6. Conclusion

In conclusion, patients prescribed gabapentin should be informed about the potential risk of mood changes, and appropriate monitoring should be implemented to mitigate the risk of developing depression.

## Author contributions

**Conceptualization:** Hao Zhang, Hua Huang.

**Data curation:** Hao Zhang, Panli Zhao, Ping Zhang, Hua Huang.

**Formal analysis:** Hao Zhang, Hua Huang.

**Investigation:** Hao Zhang, Panli Zhao, Hua Huang.

**Methodology:** Hao Zhang, Hongqi Ou, Xi Luo, Hua Huang.

**Project administration:** Hao Zhang, Hua Huang.

**Resources:** Hao Zhang, Xi Luo, Ping Zhang, Hua Huang.

**Software:** Hao Zhang, Hongqi Ou, Ping Zhang, Hua Huang.

**Supervision:** Hao Zhang.

**Validation:** Hao Zhang.

**Visualization:** Hao Zhang.

**Writing – original draft:** Hao Zhang, Hua Huang.

**Writing – review & editing:** Hao Zhang, Hua Huang.
